# Structural and regulatory diversity shape HLA-C protein expression levels

**DOI:** 10.1038/ncomms15924

**Published:** 2017-06-26

**Authors:** Gurman Kaur, Stephanie Gras, Jesse I. Mobbs, Julian P. Vivian, Adrian Cortes, Thomas Barber, Subita Balaram Kuttikkatte, Lise Torp Jensen, Kathrine E. Attfield, Calliope A. Dendrou, Mary Carrington, Gil McVean, Anthony W. Purcell, Jamie Rossjohn, Lars Fugger

**Affiliations:** 1 MRC Human Immunology Unit, Weatherall Institute of Molecular Medicine, John Radcliffe Hospital, University of Oxford, Oxford OX3 9DS, UK; 2 Infection and Immunity Program and the Department of Biochemistry and Molecular Biology, Biomedicine Discovery Institute, Monash University, Clayton, Victoria 3800, Australia; 3 Australian Research Council Centre of Excellence in Advanced Molecular Imaging, Monash University, Clayton, Victoria 3800, Australia; 4 Oxford Centre for Neuroinflammation, Nuffield Department of Clinical Neurosciences, Division of Clinical Neurology, John Radcliffe Hospital, University of Oxford, Oxford OX3 9DS, UK; 5 Wellcome Trust Centre for Human Genetics, University of Oxford, Oxford OX3 7BN, UK; 6 Department of Clinical Medicine, Aarhus University Hospital, 8200N Aarhus, Denmark; 7 Cancer and Inflammation Program, Leidos Biomedical Research Inc., Frederick National Laboratory for Cancer Research, Frederick, Maryland 21702, USA; 8 The Ragon Institute of MGH, MIT and Harvard, Cambridge, Massachusetts 02139, USA; 9 Big Data Institute, Li Ka Shing Centre for Health Information and Discovery, University of Oxford, Oxford OX3 7FZ, UK; 10 Institute of Infection and Immunity, Cardiff University, School of Medicine, Heath Park, Cardiff CF14 4XN, UK

## Abstract

Expression of HLA-C varies widely across individuals in an allele-specific manner. This variation in expression can influence efficacy of the immune response, as shown for infectious and autoimmune diseases. MicroRNA binding partially influences differential HLA-C expression, but the additional contributing factors have remained undetermined. Here we use functional and structural analyses to demonstrate that HLA-C expression is modulated not just at the RNA level, but also at the protein level. Specifically, we show that variation in exons 2 and 3, which encode the α1/α2 domains, drives differential expression of HLA-C allomorphs at the cell surface by influencing the structure of the peptide-binding cleft and the diversity of peptides bound by the HLA-C molecules. Together with a phylogenetic analysis, these results highlight the diversity and long-term balancing selection of regulatory factors that modulate HLA-C expression.

## Abstract (web summary)

HLA-C expression levels correlate with immune responses to pathogens and autoimmunity, and vary in an allele-specific manner across individuals. Here the authors identify factors that drive differential expression of HLA-C allomorphs at the cell surface, and influence the structure of the peptide-binding cleft and diversity of peptides bound by HLA-C molecules.

The human leukocyte antigen (*HLA*) gene locus is one of the most diverse regions of the human genome, with extreme polymorphism and associations with a large number of human diseases^1^. HLA molecules have diverse clinical implications in infectious and autoimmune diseases, cancer, transplantation and in pregnancy^2^,^3^. While antigenic specificity is important in dictating the immune response driven by the HLA molecule, HLA protein levels at the cell surface also play an important role in controlling the strength of the immune response^4^,^5^. Indeed, cytokine-driven upregulation of cell surface HLA in an acute infection highlights the importance of HLA expression levels in mediating host defence against pathogens^6^.

HLA class I molecules, encoded by *HLA-A*, *HLA-B* and *HLA-C*, are highly polymorphic and can bind and present a range of intracellular peptides to cytotoxic CD8+ T cells, as well as regulate innate immune responses by interacting with killer cell immunoglobulin-like receptors (KIR) expressed on natural killer (NK) cells^2^,^3^. While much is known regarding the role of HLA-A and HLA-B molecules in protective and aberrant immunity, comparatively little is known about HLA-C. Compared to its counterparts, HLA-C is expressed at lower cell surface levels, is less polymorphic, and has evolved to have more extensive interactions with KIRs, thereby playing a key role in regulating NK cell responses^7^,^8^,^9^,^10^.

HLA-C expression varies widely in an allele-specific manner^4^,^11^ and this diversity is an important determinant in influencing disease outcome, especially as observed in the case of HIV-1 infection^4^,^12^,^13^,^14^. Thus, high HLA-C protein expression in the host has been associated with protection against the HIV-1 virus, increased cytotoxic T lymphocyte responses and increased frequency of viral escape mutations, suggesting that higher HLA-C expression exerts a selection pressure on the virus^4^, which is in line with the recently discovered virus-mediated downregulation of HLA-C expression^15^. In contrast, high HLA-C expression levels correlate with increased risk of Crohn’s disease^4^,^11^, and in cases of unrelated haematopoietic transplantation, with poor outcome and graft-versus-host disease^5^. The divergent effects of HLA-C expression on infectious and autoimmune diseases, combined with evidence for the recent origin of mutations that influence expression^16^, suggest a dynamic evolutionary balance between positive and negative gene regulation, which can shift with the epidemiological cycling of specific pathogens.

There has been a wide interest in identifying factors that influence differential expression of HLA-C molecules. A single nucleotide polymorphism 35 kb upstream of *HLA-C* (-35 C/T) was correlated with HIV-1 viral load and HLA-C expression in people of European ancestry^12^,^14^,^17^. However, it was subsequently shown that this variant was not causative, and was in linkage disequilibrium with another variant in the 3′ untranslated region (UTR) of *HLA-C*, which is a polymorphic microRNA binding site for miR-148a. *HLA-C* alleles that have an intact miR-148a binding site, such as C*07 and C*03 among others, have low expression as a result of inhibition by the microRNA, whereas other *HLA-C* alleles (for example, C*05, C*08) that escape miR-148a binding due to a deletion in the miR binding site, are expressed at higher levels. This insertion/deletion polymorphism in the 3′UTR of *HLA-C* is only fractionally responsible for the differential surface expression of *HLA-C* alleles^13^. Variation in miR-148a expression itself has also been shown to further influence HLA-C levels. However, this still does not fully account for the variation in expression of *HLA-C* alleles with an intact miR-148a binding site, and has no impact on those alleles that escape miR-148a regulation^11^. Alleles of *HLA-C* show a continuous rather than a bimodal expression pattern, suggesting that additional factors with stronger effects than the miR binding site are primarily responsible for influencing differential HLA-C surface expression^13^.

To further understand the mechanisms responsible for differential HLA-C expression, we chose two *HLA-C* alleles, C*05 and C*07, which are commonly found at allele frequencies ranging between 3–14% (C*05) and 18–38% (C*07) in Caucasian populations^18^, have high and low expression, respectively, and differ in the 3′UTR miR-148a binding site variant. Using a series of functional and structural analyses, we show that variation in exons 2 and 3, which encode the antigen-binding α1 and α2 domains of HLA-C molecules, contributes to differential cell surface expression of these *HLA-C* allomorphs. This regulation is found to be post-transcriptional as the differential cell surface expression does not correlate with mRNA levels. Furthermore, we observe that HLA-C*07 has a deeper and narrower antigen-binding cleft than the relatively flat peptide-binding cleft of HLA-C*05. In line with this, HLA-C*05 binds a larger range of peptides than HLA-C*07, which can stabilize it on the cell surface, hence offering a potential explanation for the differential cell surface expression of these *HLA-C* allomorphs.

## Results

### Differential expression of *HLA-C* alleles

**Figure 1:**
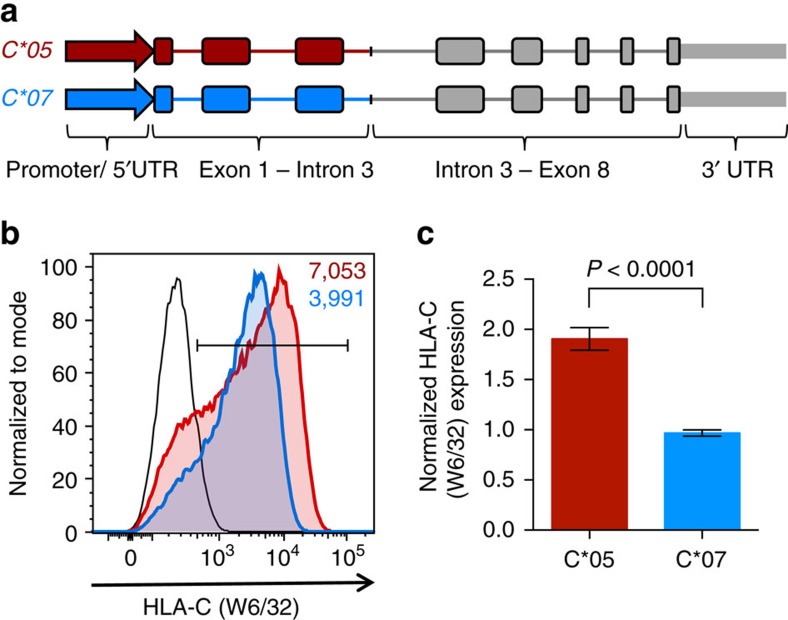
Differential expression of HLA-C*05 and HLA-C*07. (**a**) Schematic representation of C*05 (red) and C*07 (blue) genomic constructs; construct design is detailed in the methods, murine *H-2K*^*b*^ gene is shown in grey. (**b**) Representative cell surface expression of HLA-C on 721.221 cells transfected with the C*05 and C*07 genomic constructs. HLA-C (W6/32) staining is shown on GFP+ cells. C*05 (red), C*07 (blue) and vector transfected cells (black) are shown. Numbers denote mean fluorescence intensity (MFI) of HLA-C+GFP+ cells. (**c**) Normalized HLA-C (W6/32) expression on GFP+ C*05 and C*07 transfected 721.221 cells. MFI of W6/32 on the gated HLA-C+GFP+ population/MFI of GFP on GFP+ cells is plotted, and shown relative to C*07 transfected cells. Mean±s.e.m. is depicted, *n*=9.

To investigate the mechanisms responsible for differential expression of *HLA-C* molecules, we selected two common *HLA-C* alleles, HLA-C*05:01:01:01 (referred to as C*05) and HLA-C*07:02:01:03 (referred to as C*07), that differ in expression levels, and have either a disrupted (C*05) or intact (C*07) miR-148a binding site, respectively. To study the involvement of the different parts of the *HLA-C* gene in contributing towards differential surface expression, we generated hybrid C*05 and C*07 genomic constructs. One half of these hybrid constructs, consisting of the promoter, 5′UTR, exons 1–3 and introns 1, 2 and part of intron 3, was taken from the HLA-C*05 or HLA-C*07 alleles, while the second half of the constructs were identical, and consisted of part of intron 3, exons and introns 4–8, and the 3′UTR of the murine *H-2K*^*b*^ allele (Fig. 1a); this allowed us to exclude the involvement of the miR-148a binding in differential HLA-C expression levels. Importantly, similar hybrid constructs for other HLA class I genes have been described before, and shown to retain the peptide-binding specificity of the HLA allele^19^. The C*05 and C*07 hybrid constructs were transfected into HLA class I-negative 721.221 cells along with a GFP plasmid to control for transfection efficiency, and the level of HLA-C surface expression on transfected cells was determined by flow cytometry. We observed a ∼2-fold higher expression of HLA-C*05 on the cell surface of transfected cells, in comparison to cells that expressed HLA-C*07 (Fig. 1b,c and Supplementary Fig. 1a). This relative expression difference between C*05 and C*07 transfected cells was physiologically relevant as it was comparable to the relative difference in expression between HLA-C*05 and HLA-C*07 on human peripheral blood lymphocytes, which is reported to be between 1.5 and 2-fold^4^. This was of particular interest considering that both our hybrid constructs had an identical 3′UTR, as well as a region starting from a part of intron 3 until, and including, exon 8. These findings therefore indicated that variations either in the promoter, 5′UTR, exons 1–3 (which includes the peptide-binding cleft) or introns 1–3, of HLA-C*05 and HLA-C*07 were contributing to the differential HLA-C expression.

### Influence of the promoter/5′UTR of *HLA-C* on gene expression

**Figure 2:**
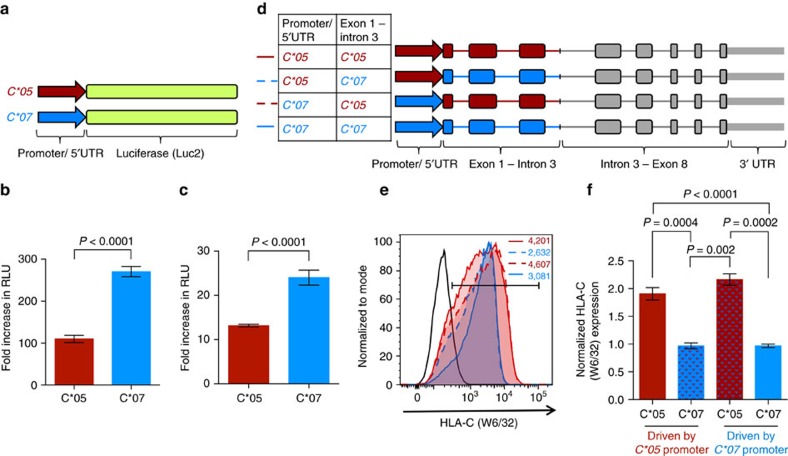
The promoter/5′UTR of HLA-C*05 and HLA-C*07 affects expression of the luciferase reporter gene but not the differential cell surface expression of HLA-C. (**a**) Schematic representation of the luciferase reporter constructs; construct design is detailed in the methods. Luciferase reporter constructs were transfected into (**b**) HEK 293 T cells, and (**c**) 721.221 cells, and dual luciferase reporter assays performed on cell lysates. Relative light units (RLU) plotted as fold change in luciferase activity of the promoter/5′UTR of the *HLA-C* alleles compared to empty-vector is shown. (**d**) Schematic representation of the C*05 and C*07 genomic constructs with or without the swapped promoter/5′UTR. (**e**) Representative cell surface expression of HLA-C on 721.221 cells transfected with the C*05 and C*07 genomic constructs. HLA-C (W6/32) staining is shown on GFP+ cells. Histogram colour coding is indicated in **d**, black line represents vector-transfected cells, numbers denote MFI. (**f**) Normalized HLA-C (W6/32) expression on GFP+ C*05 and C*07 transfected 721.221 cells. MFI of W6/32 on the gated HLA-C+ GFP+ population/MFI of GFP on GFP+ cells is plotted, and shown relative to C*07 transfected cells. Mean±s.e.m. is depicted, (**b**) *n*=12, (**c**) *n*=9, (**e**,**f**) *n*=6–9.

To test whether the promoter/5′UTRs of the HLA-C*05 and HLA-C*07 alleles were driving the protein-level differences that we observed, we cloned their promoter/5′UTR sequences (including a region 776 or 766 bp before the start codon respectively) upstream of the luciferase gene in a promoter-less vector (Fig. 2a). HEK 293T cells and 721.221 cells were transfected with these C*05-luciferase and C*07-luciferase constructs and relative luciferase activity was measured. Surprisingly, the promoter/5′UTR of C*07 led to a significantly (∼2-fold) higher expression of the luciferase reporter gene in comparison to the C*05 promoter (Fig. 2b,c)—an effect which was in the opposite direction of what was observed on the cell surface of C*05- and C*07-transfected cells. This differential effect of the promoter/5′UTR of C*07 on luciferase expression has been evidenced in a previous study, that included the region of its core promoter, and reported that the core promoter of C*07 was significantly more active than its C*06 counterpart^20^. To assess how the promoter directly influenced expression of the HLA-C molecules, we swapped the promoter/5′UTR of C*05 and C*07, and generated new hybrid constructs (Fig. 2d), which were transfected into 721.221 cells. Swapping of the promoters did not result in a change in cell surface expression of C*05 and C*07: C*05 was consistently expressed at higher levels (∼2-fold relative to C*07) on the cell surface, irrespective of the promoter/5′UTR driving its transcription (Fig. 2e,f and Supplementary Fig. 1b). Thus, despite having a seemingly weaker promoter/5′UTR region, the cell surface protein levels of HLA-C*05 remained significantly higher as compared to HLA-C*07. As the variation in the promoter/5′UTR of these alleles could not explain their differential protein expression, this inferred that the relevant region was between exons and introns 1–3.

### Variation in exons 2 and 3 affects HLA-C expression

**Figure 3:**
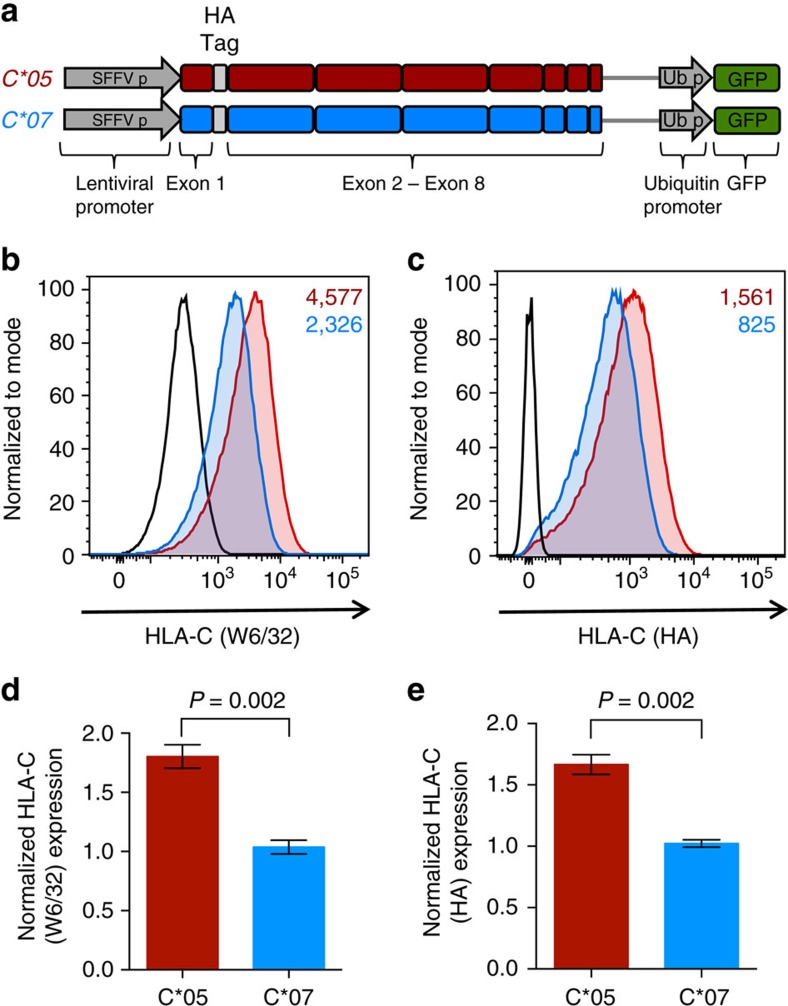
Lentiviral expression of HLA-C*05 and HLA-C*07 using exonic constructs preserves the expression pattern of HLA-C molecules. (**a**) Schematic representation of the HA-tagged C*05 and C*07 lentiviral constructs which include the sequence of exons 1–8 from the respective *HLA-C* alleles; HLA-C expression is driven by a common SFFV lentiviral promoter. Representative cell surface expression of HLA-C on 721.221 cells transduced with the lentiviral C*05 and C*07 constructs. (**b**) HLA-C (W6/32) staining and (**c**) HLA-C (HA) staining is shown on GFP+ cells. C*05 (red), C*07 (blue) and vector transduced cells (black) are shown, numbers denote MFI. (**d**) Normalized HLA-C (W6/32) expression and (**e**) HLA-C (HA) expression on GFP+ C*05 and C*07 transduced 721.221 cells. MFI of W6/32 or HA/MFI of GFP, on the GFP+ population is plotted, and shown relative to C*07 transduced cells. Mean±s.e.m. is depicted, *n*=6.

**Figure 4:**
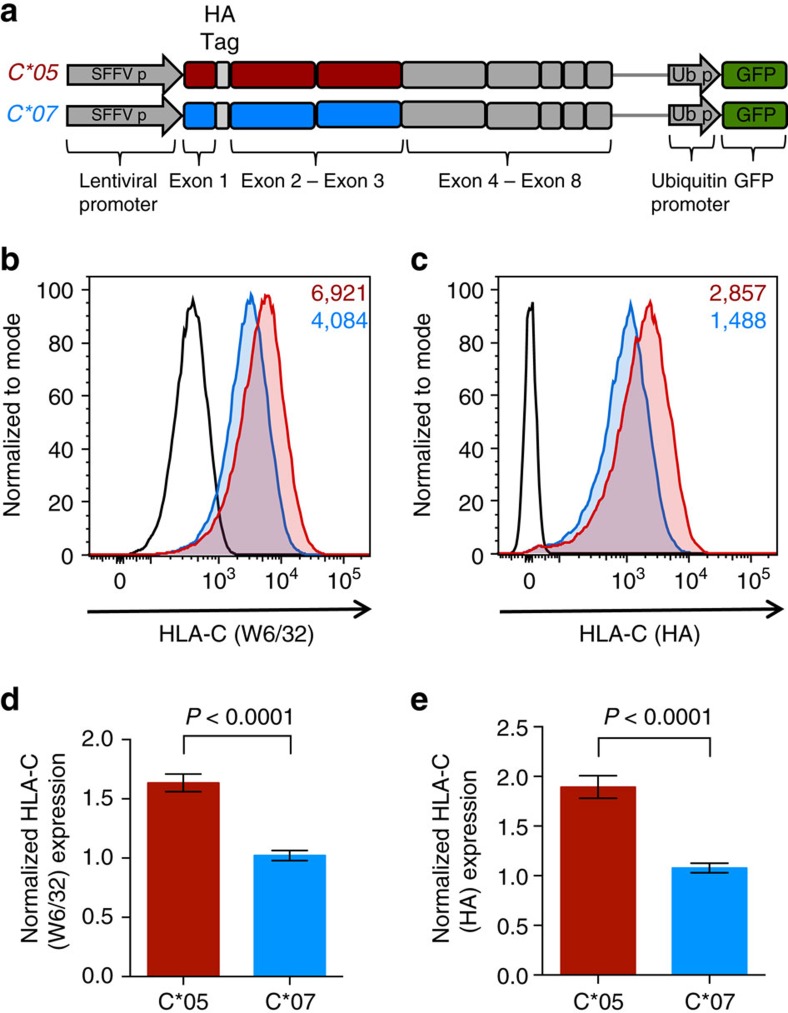
Variation in exons 2–3 (α1/α2 domains) of *HLA-C* is responsible for differential expression of C*05 and C*07. (**a**) Schematic representation of the modified HA-tagged C*05 and C*07 lentiviral constructs which include the sequence of exons 1–3 from the respective *HLA-C* alleles, and sequence of exons 4–8 of the murine *H-2K*^*b*^ gene; HLA-C expression is driven by a common SFFV lentiviral promoter. Representative cell surface expression of HLA-C on 721.221 cells transduced with the modified lentiviral C*05 and C*07 constructs. (**b**) HLA-C (W6/32) staining and (**c**) HLA-C (HA) staining is shown on GFP+ cells. C*05 (red), C*07 (blue) and vector transduced cells (black) are shown, numbers denote MFI. (**d**) Normalized HLA-C (W6/32) expression and (**e**) HLA-C (HA) expression on GFP+ C*05 and C*07 transduced 721.221 cells. MFI of W6/32 or HA/MFI of GFP, on the GFP+ population is plotted, and shown relative to C*07 transduced cells. Mean±s.e.m. is depicted, *n*=9–11.

To specifically investigate if the exonic coding region of *HLA-C* could have a direct effect on HLA-C expression levels, we used a lentiviral expression system, where the expression of the coding region of C*05 and C*07 was driven by a common lentiviral promoter. Although the anti-HLA antibody (W6/32) that we used to stain for HLA-C expression has monomorphic specificity and binds fully assembled HLA class I molecules with equal affinity^21^,^22^, we included an N-terminal hemagglutinin (HA) tag in these constructs as an additional control. To establish the validity of the system, HA-tagged C*05 and C*07 constructs including the sequence of exons 1–8 of these alleles were generated (Fig. 3a), and 721.221 cells were transduced with the respective C*05 and C*07 lentivirus at equivalent multiplicity of infection (normalized using GFP, expressed in tandem from the lentiviral expression vector). The differential expression pattern of HLA-C, detected on the cell surface by the anti-HLA (Fig. 3b,d), and anti-HA (Fig. 3c,e) antibodies, was preserved in these lentiviral-transduced cells at levels similar to those observed with the transiently transfected cells (Figs 1c and 2f). Importantly, the expression difference between C*05 and C*07 was seen to be consistent between HA-tagged and non-tagged *HLA-C* constructs, suggesting that the HA tag, itself, does not change the HLA-C expression pattern or cellular characteristics, also shown by a previous study comparing HA-tagged and non-tagged HLA class I molecules^23^. To then test whether variation in α1 and α2 domains of the HLA-C molecules was responsible for the differential expression, we generated modified lentiviral expression constructs that contained only exons 1–3 of the C*05 and C*07 alleles and exons 4–8 of the murine *H-2K*^*b*^ allele (Fig. 4a). Interestingly, cell surface staining revealed a significant and consistently high expression (∼1.7-fold) of C*05 in comparison to C*07 in 721.221 cells transduced with the modified lentivirus, demonstrating that variation in the α1/α2 domains of HLA-C was controlling the differential HLA-C expression (Fig. 4b–e). Furthermore, this appeared to be a post-transcriptional event, as no significant changes in *HLA-C* were observed at the mRNA level, as tested using exon-spanning QPCR primers designed for an *H-2K*^*b*^region common to both *HLA-C* constructs (Supplementary Fig. 2). Additionally, staining for HLA-C after fixation and permeabilization of transduced cells (Supplementary Figs 3 and 4), or quantification of HLA-C protein levels by immunoblotting of whole cell lysates (Supplementary Figs 5 and 6), did not reveal a difference in total protein-level expression between C*05 and C*07, despite the cell-surface difference (Figs 3 and 4). This may be related to accumulation and retention of HLA-C folding intermediates inside the cell, before successful peptide loading and export to the cell surface, such that total protein expression is unaffected but there is a differential expression level at the plasma membrane^24^,^25^. Taken together, these data demonstrate that variation in the coding region of HLA-C, specifically the α1 and α2 domains, can drive differential HLA-C expression at the cell surface.

### HLA-C*05 and C*07 have contrasting antigen-binding clefts

To elucidate the role of α1/α2 domains and the peptide-binding cleft of HLA-C on differential expression, we solved the structure of HLA-C*05 in complex with a HLA-C*05 specific peptide, SAEPVPLQL (SAE)^26^, and HLA-C*07 in complex with a HLA-C*07 specific peptide, RYRPGTVAL (RYR)^27^ (Supplementary Table 1).

**Figure 5:**
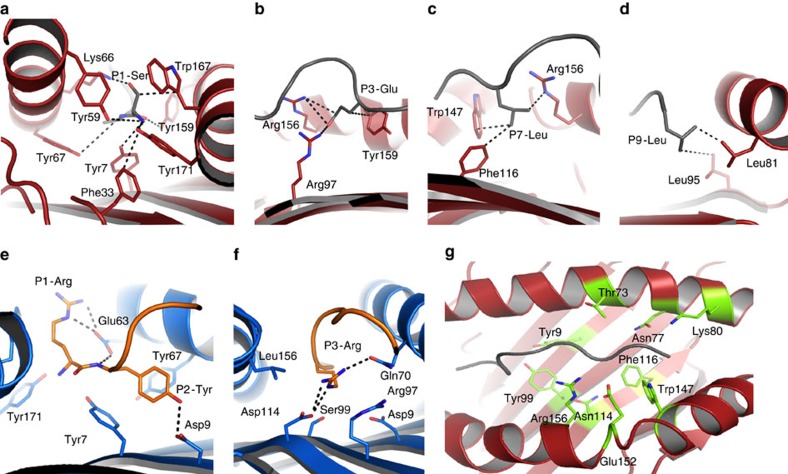
Peptide-HLA-C interactions. (**a**–**d**) represent the interaction of the HLA-C*05 molecule (red) with the SAE peptide (grey), with the residues involved in the interaction represented as sticks. (**e**,**f**) represent the interaction of the HLA-C*07 molecule (blue) with the RYR peptide (orange). The black dashed lines represent the interaction between the peptide and HLA molecule. (**g**) HLA-C*05 α1/α2 domains structure represented in cartoon (red) with the polymorphic residues that differ with HLA-C*07 coloured in green.

Within the HLA-C*05-SAE complex, there are four main anchor residues, P1-Ser, P3-Glu, P7-Leu and P9-Leu. The P1-Ser is surrounded by seven aromatic residues, arising from the floor of the antigen-binding cleft (Tyr7, Tyr67 and Phe33) and from the α1 and α2 helices (Tyr59, Tyr171, Tyr159 and Trp167), as well as hydrogen bonding to Lys66 (Fig. 5a). The P3-Glu forms a salt bridge with Arg156 and Arg97, and has a hydrophobic interaction with Tyr159 (Fig. 5b). In addition, the P7-Leu places its hydrophobic side chain underneath Arg156 and binds within a hydrophobic pocket lined by Phe116 and Trp147 (Fig. 5c). Finally, the P9-Leu is anchored in the F pocket of HLA-C*05 and interacts with the 2 hydrophobic residues, Leu81 and Leu95 (Fig. 5d).

The structure of HLA-C*07 in complex with the RYR peptide revealed canonical P2-Tyr and P9-Leu anchor residues, a large network of interactions at P1-Arg and a secondary anchor residue at P3-Arg. The P1-Arg in HLA-C*07 was stabilized by aromatic residues, similarly to the P1-Ser of the SAE peptide in HLA-C*05, with an additional salt bridge formed with the Glu63 (Fig. 5e). The large P2-Tyr sat into the B pocket, stabilized by a hydrogen bond with Asp9 and van der Waals interactions with Tyr7 and Tyr67 (Fig. 5e). In contrast to HLA-C*05, the B pocket of HLA-C*07 is deeper due to the smaller polymorphic residue at position 9, which is Asp in HLA-C*07, as opposed to Tyr in HLA-C*05. The P3-Arg of the RYR peptide, which was buried within the antigen-binding cleft, acted as a secondary anchor residue when binding to the HLA-C*07 molecule. The absence of the Arg156 in HLA-C*07 (replaced with a smaller and hydrophobic Leu156) allowed the P3-Arg of the RYR peptide to fit inside the cleft of the HLA-C*07 molecule (Fig. 5f). The buried conformation of the P3-Arg is facilitated by the presence of small residues at position 9 (Asp→Tyr) and 99 (Ser→Tyr) in the cleft of HLA-C*07 that allowed enough space for the Arg97 to move away from the P3-Arg. P3-Arg is stabilized by a hydrogen bond with the Gln70 and salt bridge with Asp114 (Fig. 5f).

**Figure 6:**
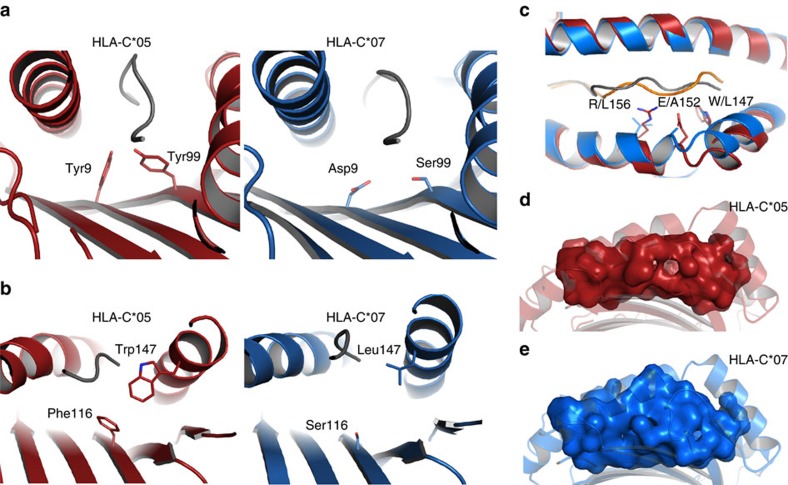
Structural comparison of HLA-C*05 and HLA-C*07. (**a**,**b**) represent the HLA-C*05 structure (red) or the HLA-C*07 (blue) based on the HLA-C*05 structure in the same orientation. (**c**) shows the superposition of the HLA-C*05 and HLA-C*07 structures, coloured as red and blue, respectively. (**d**,**e**) show a surface representation of the antigen-binding cleft of HLA-C*05 (red) and of the HLA-C*07 (blue), calculated using the CASTp web server^63^.

HLA-C*05 and HLA-C*07 differ by 22 residues, of which 15 are located within the α1/α2 domains, with ten of these being involved in peptide interactions, namely Tyr9, Thr73, Asn77, Lys80, Tyr99, Asn114, Phe116, Trp147, Glu152 and Arg156 (Fig. 5g). While HLA-C*05 uses a large network of aromatic residues in both the A and B pockets, the HLA-C*07 B pocket lacks two of these tyrosines (Tyr9→Asp9 and Tyr99→Ser99) (Fig. 6a). The presence of these smaller residues and Asp9 in HLA-C*07 is consistent with the preference of P2 Arg/Tyr for HLA-C*07-restricted peptides, as previously reported^28^. Similarly, the F pocket of HLA-C*05 was ‘filled’ by large aromatic residues (Phe116 and Trp147), which were absent from HLA-C*07 (Ser116 and Leu147) (Fig. 6b). In addition to the larger Trp147, the hinge of the α2-helix of HLA-C*05 differs from HLA-C*07 by two other large residues, namely Glu152 (Ala152 in HLA-C*07) and Arg156 (Leu156 in HLA-C*07). These large residues located on the a2-helix of HLA-C*05 open the antigen-binding cleft by almost 3Å (residues 149 to 151) (Fig. 6c), while the rest of the cleft was similar (r.m.s.d. of 0.62 Å on the Cα of the α1-α2 domains).

Overall the B and F pockets, binding the characteristic anchor residues at P2 and the C-terminus of HLA class I-restricted epitopes, contain large aromatic residues in HLA-C*05 that are absent in HLA-C*07. Consequently, the antigen-binding cleft of HLA-C*05 is composed of residues with large side chains, and accordingly offers a more shallow cleft (volume 1,200 Å^3^, Fig. 6d) in contrast to the HLA-C*07 cleft that is deeper and narrower with a larger volume (1,500 Å^3^, Fig. 6e). Therefore, the polymorphic residues are ‘filling’ the cleft of HLA-C*05 that represents a relatively shallow groove, providing a ‘peptide-landing platform’ for HLA-C*05, instead of the traditional groove generally found in HLA molecules that are more prone to have preference for specific anchoring motifs (Fig. 6d,e).

The apparent ‘flat cleft’ of HLA-C*05 might allow binding of a more diverse range of peptides, which could impact the stability of the peptide-HLA-C complexes and contribute to differential HLA-C expression at the cell surface. To test this, we refolded both HLA-C*05 and HLA-C*07 with four different peptides, including two HLA-C*05 peptides (a self-peptide, ITASRFKEL (ITA)^29^, and the viral peptide SAE^26^ and two HLA-C*07 peptides (two self peptides, RYRPGTVAL (RYR)^27^ and KYFDEHYEY (KYF)^30^), and compared the thermal stability of these peptide-HLA-C complexes. In line with the structural data, HLA-C*07 showed a preference for P2 Arg/Tyr, and its stability was 5–10 °C higher when refolded with the HLA-C*07 peptides, RYR and KYF, in comparison to its stability with the HLA-C*05 peptides, SAE and ITA. Contrastingly, the HLA-C*05 molecule exhibited the same thermal stability with the HLA-C*05 peptides as well as the HLA-C*07 peptides, with an average Tm of ∼52 °C (Supplementary Table 2). In line with the structural analyses, these results indicate that, unlike for HLA-C*07, the stability of HLA-C*05 was less reliant on the sequence of the bound peptides, and that HLA-C*05 might be more permissive than HLA-C*07 in its peptide-binding motif, which could impact its differential expression pattern.

### Comparison of the peptides bound by HLA-C*05 and HLA-C*07

To compare the peptide repertoire of HLA-C*05:01 and HLA-C*07:02, we isolated these HLA class I molecules from the cell surface of equal numbers of C*05 and C*07 transfected 721.221 cells, and sequenced bound peptides by mass spectrometry.

**Figure 7:**
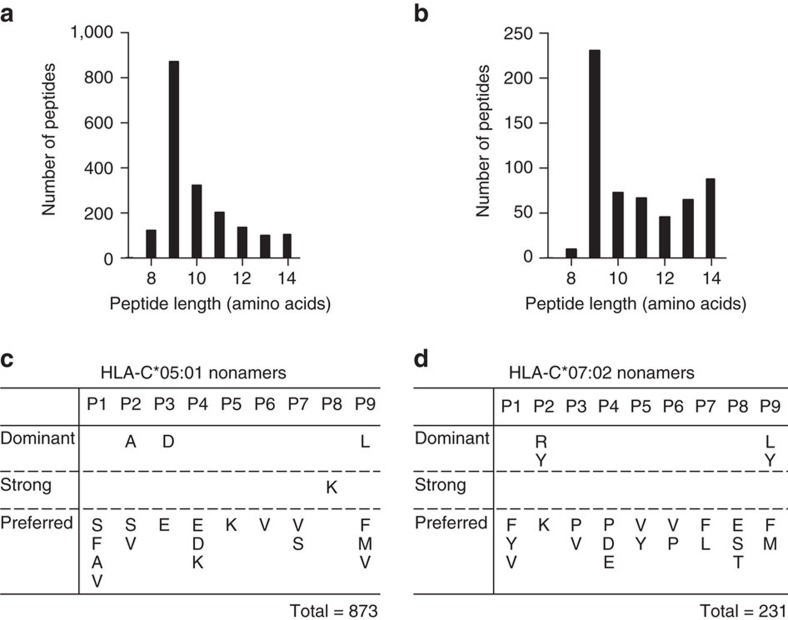
Comparison of peptide repertoire of HLA-C*05 and HLA-C*07. Peptide length analysis of (**a**) HLA-C*05:01 and (**b**) HLA-C*07:02 transfected 721.221 cells. Peptide motifs identified for nonamers for (**c**) HLA-C*05:01 and (**d**) HLA-C*07:02 are shown. Residues identified as dominant occur at a frequency of>30%, strong>20% and preferred>10%. Data were collated from three independent experiments for each allele.

A total of 1,870 specific peptides were identified from HLA-C*05 molecules (Supplementary Data 1). The majority of these peptides (70.6%) were 8–10 amino acids in length, with nonamers being the most abundant species (46.7%) (Fig. 7a). Analysis of nonameric peptides revealed three positions with conserved residues (P2, P3 and P9). The P3 position was by far the most conserved with 80% of the peptides having an Asp at this position, and a further 15% having Glu. The P2 position optimally preferred a small uncharged residue such as Ala (40%), and to a smaller extent Ser (13%) and Val (11%). At the P9 position, the majority of the peptides carried a hydrophobic residue, with 45% of the peptides carrying a Leu, with smaller contributions from Phe (17%), Met (13%) and Val (10%) (Fig. 7c).

A total of 580 specific peptides were identified from HLA-C*07 molecules (Supplementary Data File 1). The majority of the peptides observed (54.1%) were also 8–10 amino acids in length, with nonamers being the most abundant species (39.8%), however, this was less than that observed for HLA-C*05 (Fig. 7b). Analysis of nonamers revealed only two positions with conserved residues (P2 and P9). The P2 position was most conserved with Arg being most dominant (40%), closely followed by Tyr (38%), and a small contribution from Lys (13%). The P9 position preferred a hydrophobic residue with Leu (31%) being most conserved; however, HLA-C*07 also appeared to accept larger hydrophobic residues such as Tyr (30%), Phe (17%) and Met (13%) (Fig. 7d).

In line with the structural analysis, these peptide-repertoire data demonstrate that the number of distinct peptides bound by HLA-C*05 were threefold higher than those bound by HLA-C*07, in agreement with the higher relative expression of HLA-C*05 on the cell surface. Collectively, these data provide insight into how the α1/α2 domains and the peptide-binding cleft of HLA-C molecules can not only have a direct influence on HLA stability and peptide repertoire, but also influence cell surface expression levels.

### Phylogenetic analysis of *HLA-C* sequences

To assess the evolutionary origin of the variation in exons 2 and 3 of *HLA-C* alleles, which we show influences HLA-C expression levels, we performed sequence alignments of the exons 2 and 3 region of *HLA-C* alleles with the available non-human primate *MHC-C* alleles, and inferred their phylogenetic relationship (Supplementary Fig. 7). Within the exons 2 and 3 sequence, the HLA-C*07 alleles seemed more closely related to a set of chimpanzee *Patr-C* alleles, than to HLA-C*05 alleles. This indicated a maintenance of HLA-C*07-like alleles in non-human primates, whereas the HLA-C*05-like alleles have only been found in humans. As expected, there was also evidence for additional diversity, and groups of chimpanzee-specific *MHC-C* and human-specific *HLA-C* sequences.

**Figure 8:**
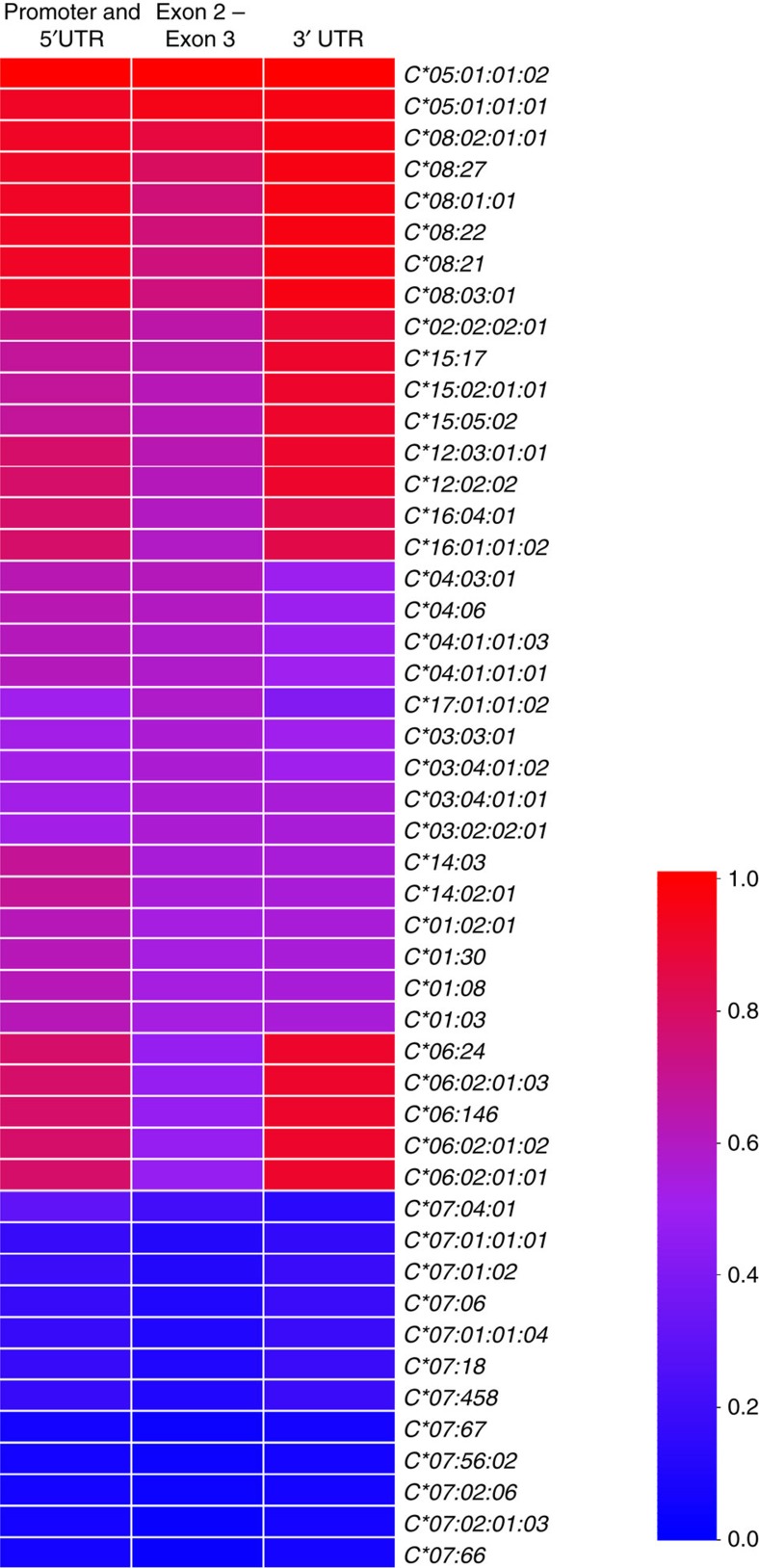
Patterns of genetic diversity in *HLA-C* alleles at three regulatory sites. Each grid represents the similarity between an *HLA-C* allele and the HLA-C*05:01:01:01 and HLA-C*07:02:01:03 alleles, and it is coloured based on its similarity to C*05. Similarity is determined through phylogenetic analysis at the promoter/5′UTR, exons 2–3, and 3′UTR regions. The display of HLA-C subgroup alleles is based on their similarity ranking in the exons 2−3 region. Inferred trees utilized to extract these similarities are presented in Supplementary Fig. 8.

Our study, combined with previous work^13^, suggests that there are three regions of *HLA-C* that have the ability to regulate differential HLA-C expression, that is, the promoter/5′UTR, exons 2 and 3, and the 3′UTR. To compare the diversity of genetic variants in these three regions, we performed phylogenetic analysis for each of these regions across a range of *HLA-C* alleles (Supplementary Fig. 8). These phylogenetic trees were then used to calculate phylogenetic distances between *HLA-C* alleles for each of these regions. Using HLA-C*05 and HLA-C*07 as references, the degree of similarity between a *HLA-C* allele and HLA-C*05 or HLA-C*07 was determined for each region, and plotted as a grid (Fig. 8). This *in silico* analysis revealed a wide range of variation in the three genetic regions that regulate HLA-C expression, which could be related to the observed continuous expression pattern of *HLA-C* alleles. For example, C*04 alleles, which have been shown to be expressed at high levels at the cell surface^4^, appear to be more similar to C*05 than to C*07 in the promoter/5′UTR, and exons 2 and 3 sequence. This is particularly interesting for C*04, as, based on binding of miR-148a in the 3′UTR of its mRNA^13^, and its similarity to C*07 in the 3′UTR, it would have been predicted to be a low-expresser (Fig. 8). These patterns of genetic diversity suggest that a combination of variants spread throughout the *HLA-C* gene region, and perhaps additional factors, all contribute towards allele-specific differential expression of HLA-C at both the transcript and protein levels.

## Discussion

In this study, we sought to understand the mechanisms that contribute to differential expression of HLA-C molecules. By using a comparison between two common *HLA-C* alleles, HLA-C*05 and HLA-C*07, we demonstrate that variation in exons 2 and 3 of *HLA-C*, that encode for the peptide-binding α1/α2 domains, contributes to differential cell surface HLA-C protein expression. While HLA-C*05 and HLA-C*07 levels remain unchanged at the transcript and total protein level, we find a significant difference in their relative cell surface expression, with HLA-C*05 being expressed at high levels on the cell surface. Using structural, thermal stability and peptide-repertoire comparisons, we demonstrate that the peptide-binding cleft of HLA-C*05 is more permissive and is filled with large aromatic residues, which is not the case for HLA-C*07. Our data demonstrate that instead of forming a groove as in HLA-C*07, the peptide-binding cleft of HLA-C*05 forms a flatter ‘peptide-landing platform’, that allows binding of a larger range of peptides, which can stabilize the HLA-C molecule, in turn affecting its expression levels on the cell surface.

We found that the promoter/5′UTR of *HLA-C*, which, in this study, spanned up to 776/766 bases upstream of the start codon, did not directly impact the differential surface expression of HLA-C alleles. This was surprising considering that the same promoter/5′UTR region differentially affected the expression of the luciferase reporter gene. A previous study suggested that an enhancer κB element in the core *HLA-C* promoter was responsible for its differential effect on the luciferase reporter; however, they did not investigate the direct effects of the core promoter on HLA-C expression levels^20^. We do not find any evidence that the region of the promoter/5′UTR of HLA-C tested in this study has any significant effect on *HLA-C* mRNA levels; however, it is feasible that elements outside of the tested sequence could impact mRNA expression of *HLA-C* alleles.

Studies on HLA molecules have largely focussed on their peptide binding specificities, while there has been limited emphasis on the regulatory mechanisms that control their differential allele expression and the ensuing functional implications. Small differences in expression level of *MHC/HLA* genes can influence response to pathogens, tumours, autoimmunity, as well as transplantation, potentially through both the acquired and innate immune response pathways^4^,^5^,^11^,^31^,^32^,^33^,^34^. Hence, even a two-fold difference that is observed between HLA-C allotypes, such as HLA-C*05 and HLA-C*07, is likely to have functional consequences in influencing the efficacy of the immune response. HLA-C is expressed at lower levels and is limited in polymorphism compared to its counterparts, HLA-A and HLA-B^7^,^8^,^9^,^10^. However, HLA-C is a prototypical KIR ligand and is important in the regulation of NK cell activity^7^. Although KIRs are capable of binding multiple HLA-C allotypes, it is plausible that differences in expression of HLA-C allotypes have a downstream influence on KIR signalling and NK cell function. The broad peptide specificity of KIRs^35^ raises the question of whether alleles such as HLA-C*05, whose stability is less reliant on the sequence of the bound peptide, are potentially better KIR ligands.

A previous study that attempted to understand the peptide-binding specificities of HLA-C molecules suggested that no conservation at P2 is observed for HLA-C*05-restricted peptides, hence allowing a greater diversity of amino acids to bind the B pocket^28^. However, the authors described that a HLA-C*05-specific peptide would have a preference for an Asp at position 3. Our structure of the HLA-C*05-SAE complex showed that P3-Glu forms a salt bridge with the polymorphic residue Arg156 (Leu156 in HLA-C*07), and a P3-Asp would be suited to interact in the same fashion. Furthermore, results from our thermal stability assay show that smaller residues, such as P3-Ala, could also be readily accommodated within HLA-C*05. Our peptide-elution data demonstrate that HLA-C*05 has a preference for a small residue at P2, which fits well with its shallow and flat peptide-binding cleft, and its ability to bind a greater number and range of peptides.

Post-transcriptional mechanisms such as inefficient peptide binding or association with chaperons such as TAP (transporter associated with peptide-loading) or tapasin have been proposed to contribute towards lower surface expression of HLA-C in comparison to HLA-A and HLA-B^24^,^25^,^36^; however, this has not yet been reported for differential surface expression of *HLA-C* alleles.

Studies of chicken MHC have reported an inverse correlation between diversity of peptide repertoire and cell surface MHC class I expression, with low expression correlating with resistance to Marek’s disease^37^,^38^. However, structural analyses of high- and low-expressing chicken MHC class I molecules importantly reveal that the width of the peptide-binding groove is large in low-expressing molecules, and narrow in high-expressing MHC class I molecules^37^,^38^,^39^. Similarly, a difference in thermal stability is correlated to surface expression levels^40^. The presentation of peptides on the cell surface of chicken MHC is reliant on the peptide-translocation specificity of TAP, which is known to vary between chicken haplotypes and by TAP polymorphism^39^,^40^; such peptide-translocation specificity of TAP is not found in humans^41^.

**Figure 9:**
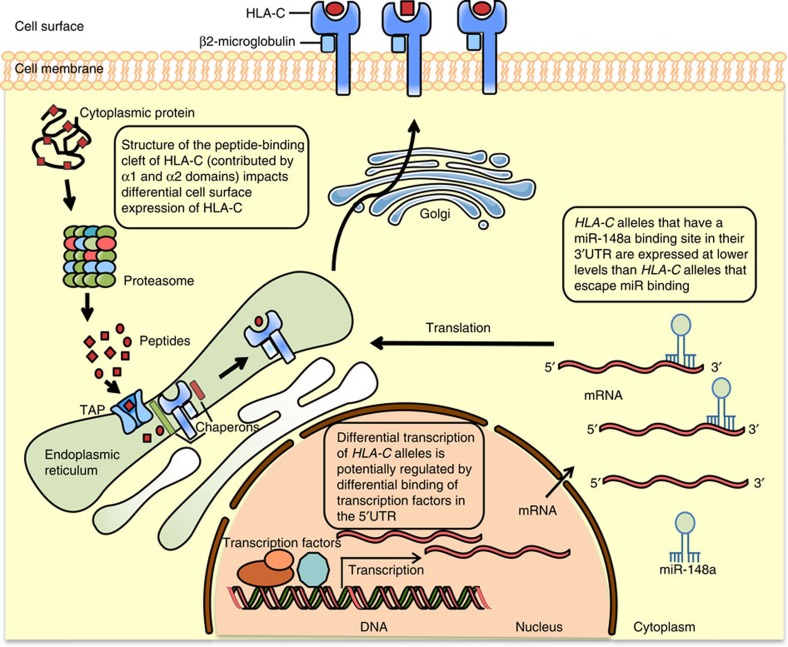
The regulatory landscape of HLA-C expression. A combination of variants in the 5′UTR, the antigen-binding cleft and the 3′UTR, and potentially other yet unidentified factors, drive differential HLA-C expression at the cell-surface. The graphics in this figure were adapted from Servier Medical Art licensed under a Creative Commons Attribution 3.0 Unported License.

Our results, combined with previous work, show that HLA-C expression is modulated by multiple factors acting at several levels, from transcription to miRNA binding and peptide selectivity mediated by the antigen-binding cleft—consequently leading to a net effect that determines abundance at the cell surface (Fig. 9). Such diversity points to a complex evolutionary history. Here, we show that the antigen-binding cleft-encoding sequence of exons 2 and 3 of C*07-like alleles has been maintained in primates for millions of years and can be found in modern populations of chimpanzees and other species, while no C*05-like alleles were found in the chimpanzee sequences available. By contrast, the 3′ miRNA binding site polymorphism seems to have arisen since the split of the human and chimpanzee ancestors, through a gene conversion event from an *HLA-B* sequence^16^. Similarly, there seems to be no evidence for shared polymorphism in the promoter region, likewise indicating that these variants have also risen since the species diverged^42^. This complex evolutionary and regulatory landscape is suggestive of an ever-changing selective regime, perhaps resulting from transient selection for up- or downregulation of specific groups of alleles with particular binding specificities, in response to particular pathogens and endogenous factors such as autoimmunity and pregnancy.

## Methods

### Transient transfection assays and constructs

The C*05 and C*07 hybrid constructs were made by amplifying∼2.04 and 2.06 kb genomic fragments of HLA-C*05:01:01:01 and HLA-C*07:02:01:03 respectively, which contained 776 or 766 bp of the respective *HLA* 5′UTR and exons 1–3 up to a midpoint in intron 3, which was fused to a∼3.58 kb fragment of the genomic *H-2K*^*b*^ gene, beginning at a midpoint in intron 3 and containing exons 4–8 and the *H-2K*^*b*^ 3′UTR. For experiments with swapped promoters/5′UTR, additional hybrid constructs were made where the 5′ flanking region of the HLA genes was interchanged, such that the HLA-C*05:01:01:01 promoter/5′UTR was fused to the exons 1–3 sequence of HLA-C*07:02:01:03, and vice versa. The region from the genomic *H-2K*^*b*^ gene was the same as described above. These hybrid constructs were transfected into 721.221 cells using optimized electroporation conditions (260 V, 1070 μF, ∞ resistance) using a Genepulser II (Bio-Rad). A limiting concentration of the pmax-GFP plasmid (Lonza) was co-transfected as a transfection control. The cells were collected 48 h post-transfection, and used for flow cytometry or RNA isolation/QPCR experiments.

### Luciferase assays

The promoters/5′UTR of HLA-C*05:01:01:01 (776 bp upstream of the start codon) or HLA-C*07:02:01:03 (766 bp upstream of the start codon) genes were cloned into a luciferase containing pGL4.14 (Promega) basic promoter-less vector. HEK 293 T or 721.221 cells were transfected with the luciferase constructs containing the 5′UTR/promoter from either C*05, C*07 or no promoter, along with the co-transfection of the Renilla luciferase vector, pGL4.74 (Promega), using TransIT 2020 transfection reagent (for HEK 293T cells) or optimized electroporation conditions (for 721.221 cells). Cells were lysed after 24 h (HEK 293T) or 5 h (721.221) post-transfection, and firefly and renilla luciferase activities were measured using dual luciferase reporter assay system (Promega) and the Glomax multi detection system (Promega). The firefly luciferase activity was normalized relative to Renilla luciferase for each transfection, and the luciferase activity of each reporter construct was calculated as a fold change relative to the activity of pGL4.14-basic vector lacking a promoter.

### Lentiviral expression assays

The C*05 and C*07 lentiviral expression constructs were made by amplifying the cDNA of HLA-C*05:01:01:01 and HLA-C*07:02:01:03 genes from exons 1–8, and cloning it into the pHRsinUbEm expression plasmid (a gift from J.M. Boname/P.J. Lehner, University of Cambridge), with the inclusion of the HA tag at the N-terminus of C*05 and C*07, just after the signal peptide sequence. For the modified C*05 and C*07 lentiviral expression constructs, the cDNA of exons 1–3 from the respective *HLA* genes was fused to the cDNA of exons 4–8 of the murine *H-2K*^*b*^ gene, and cloned into the pHRsinUbEm expression plasmid, with inclusion of the N-terminal HA tag. The lentiviral HLA expression plasmids were co-transfected with the vesicular stomatitis virus-G envelope plasmid pMD2.G (Addgene) and packaging plasmid psPAX2 (Addgene), containing HIV-1 Gag and Rev, into HEK 293T cells to package lentiviral particles. Viral titres were determined by serial dilution and transduction of HEK 293T cells. 721.221 cells were transduced with the packaged lentivirus at a multiplicity of infection of 20, in the presence of polybrene (Santa Cruz Biotechnology), added at a final concentration of 8 μg ml^−1^. Cells were collected 72 h post transduction and flow cytometry, RNA isolation/QPCR or immunoblotting experiments were performed.

### Flow cytometry and antibodies

HLA-C cell surface expression was measured in transfected or transduced cells using alexa fluor 647 anti-human HLA-A, B, C antibody (clone W6/32, BioLegend, 311414, 1:20) or anti-HA.11 antibody (clone 16B12, Covance, MMS-101R, 1:250) along with allophycocyanin goat anti-mouse Ig (BD Biosciences, 550826, 1:50). HLA-C or HA staining was determined on GFP+ cells. For intracellular staining, cells were fixed and permeabilized using BD Cytofix/Cytoperm Fixation/Permeabilization kit (BD Biosciences), followed by staining using phycoerythrin anti-human HLA-A, B, C antibody (clone W6/32, BioLegend, 311406, 1:20) or anti-HA.11 antibody (clone 16B12, Covance, MMS-101R, 1:250) along with allophycocyanin goat anti-mouse Ig (BD Biosciences, 550826, 1:50). HLA staining using the same antibodies on non-transfected cells was used as a negative control. Data obtained were analysed using the FlowJo software (Tree Star).

### RNA isolation and real-time quantitative PCR analysis

Total RNA was isolated from cells using the RNeasy isolation kit (Qiagen), and cDNA prepared using the Quantitech Reverse Transcription kit (Qiagen). SYBR-green based quantitative PCR assays were designed and optimized for the *H-2K*^*b*^ gene (which formed a common region of the *HLA-C* constructs), *GFP* (for detecting Emerald GFP in lentiviral expression plasmids), *Copgreen* (for detecting GFP in pmaxGFP) and *UBC*. The sequences of the primers used for these assays were as follows:

*H2Kb*: 5′-GAGCTGCAATAGTCACTGGAG-3′ and 5′-CCATCACTTTACAATCTGGGAGAG-3′;

*GFP*: 5′-AAGAACGGCATCAAGGTGAAC-3′ and 5′-CAGGTAGTGGTTGTCGGG-3′;

*Copgreen*: 5′-AGGACAGCGTGATCTTCACC-3′ and 5′-CTTGAAGTGCATGTGGCTGT-3′;

*UBC*: 5′-AACATCCAGAAAGAGTCCACC-3′ and 5′- CTTGTCTTGGATCTTTGCCTTG-3′.

Standard curves for each of the assays were performed using serial dilutions of cDNA and amplification efficiencies were determined. Relative expression was expressed as 2^−dCt^, where dCt is the difference of the cycle threshold between the transcript of the gene of interest and the reference gene transcript.

### Immunoblotting

Whole cell lysates were prepared in NP-40 lysis buffer containing 150 mM NaCl, 1% NP-40 (Igepal CA-630), 50 mM Tris pH 8.0, supplemented with protease inhibitors (Roche). Protein concentration of cell lysates was determined using Pierce BCA Protein Assay Kit (Thermo Scientific) and equal amount of protein was loaded onto denaturing polyacrylamide gels. HA-tagged HLA-C protein was visualized using anti-HA.11 (clone 16B12; Covance) and normalized using anti-GFP (Life technologies) and anti-GAPDH (clone 14C10; Cell Signaling Technology) antibodies. Membranes were stained with IRDye 800CW goat-anti-mouse IgG and IRDye 680LT goat-anti-rabbit IgG secondary antibodies and visualized and quantified using an Odyssey Infra-Red Imaging System (LI-COR Biosciences).

### Statistical analysis

Statistical tests were performed using GraphPad Prism and non-parametric Mann–Whitney *U*-tests were used for comparing two experimental groups, with a 5% significance level. For swapped promoter analyses, a Bonferroni correction for multiple testing was used; considering *P*=0.05 for four independent hypotheses, the significance threshold used for this analysis was *P*=0.0125.

### Protein expression and thermal stability assays

Soluble class I heterodimers of HLA-C*05 and HLA-C*07 heavy chain and full-length β2-microglobulin (β2m) were expressed in *Escherichia coli* as inclusion bodies as previously described^43^. Both HLA molecules were refolded with four peptides ITASRFKEL, SAEPVPLQL, RYRPGTVAL and KYFDEHYEY and thermal stability assay was performed. The fluorescent dye Sypro orange was used to monitor the protein unfolding. The thermal stability assay was performed in the Real Time Detection system (Corbett RotorGene 3000), originally designed for PCR. Each pHLA complex was in 10 mM Tris-HCl pH 8, 150 mM NaCl, at two concentrations (5 and 10 μM) in duplicate, was heated from 25 to 95 °C with a heating rate of 1 °C per min. The fluorescence intensity was measured with excitation at 530 nm and emission at 555 nm. The Tm, or thermal melt point, represents the temperature for which 50% of the protein is unfolded.

### Crystallization and structure determination

Crystals of the HLA-C*05-SAE were grown by the hanging-drop, vapour-diffusion method at 20 °C with a protein/reservoir drop ratio of 1:1, at a concentration of 3 mg ml^−1^ in 10 mM Tris-HCl pH 8, 150 mM NaCl using 1.8 M Na malonate pH 7. The HLA-C*05:01-SAE crystals were flash frozen in liquid nitrogen. Crystals of HLA-C*0702-RYR were grown in 0.1 M HEPES pH 8.5, 2 mM ZnSO_4_ and 28% jeffamine ED-2001 (Hampton), using the same technique as for the HLA-C*05-SAE crystals. The HLA-C*07:02-RYR crystals were soaked in a mother liquid solution with the addition of 25% ethylene glycol prior to be flash frozen in liquid nitrogen. The data were collected on the MX1 beamline at the Australian Synchrotron^44^, using the ADSC-Quantum 210 CCD detector (at 100 K). Data were processed using the XDS^45^ and scaled using SCALA software^46^ from the CCP4 suite^47^. The structures were determined by molecular replacement using the PHASER^48^ program with the HLA-C*08 for the MHC model without the peptide (Protein Data Base accession number, 4NT6 (ref. 49)). Manual model building was conducted using the Coot software^50^ followed by maximum-likelihood refinement with the Buster program^51^ or Refmac form the CCP4 suite^47^. The final models have been validated using the Protein Data Base validation website and the final refinement statistics are summarized in supplementary table 1. All molecular graphics representations were created using PyMol^52^.

### Peptide elution

The HLA class-I negative 721.221 cells stably transfected with HLA-C*05:01 or HLA-C*07:02 were utilized to obtain the peptide repertoires in a previously described manner^53^,^54^. In short, HLA class I were purified from a total 4 × 10^9^ 721.221 cells for each HLA-C using the pan class I antibody W6/32 immobilized and cross-linked to protein A resin. Captured HLA-peptide complexes were eluted with 10% acetic acid. The dissociated complexes were further separated by Reversed-phase HPLC to isolate and fractionate the bound peptides before analysis with a Q Exactive Hybrid Quadrupole-Orbitrap Mass Spectrometer (Thermo Scientific)^54^. Peptides were identified by database search using the human UniProtKB/SwissProt database (Feb 2016) with ProteinPilot V5.0 (SCIEX). A false discovery rate of 5% was applied and known contaminant peptides removed from the final list of peptide ligands. Peptides of 8–14 amino acids in length were then used for analysis.

### Phylogenetic analysis of *HLA-C* and chimpanzee *MHC-C* alleles

Nucleotide sequences for *HLA-C* and chimpanzee *MHC-C* alleles were obtained from the IMGT database^55^. All chimpanzee sequences in the database were considered and a subset of human sequences was selected in each allele subclass. The region of the sequences aligned included exons 2 and 3 region (without the intron) and spanned 546 bp for the C*05 and C*07 sequences. Sequences were aligned with MUSCLE (v3.8.31) (ref. 56) and the exons 2 and 3 sequences were extracted from the alignment. MrBayes (v3.2.5) (ref. 57) was used to infer the phylogenetic relationship between sequences using the GTR model of nucleotide evolution with rate gamma distributed, running parameters used were nruns=3, nchains=4, ngen=2000000 and samplefreq=1000.

### Heat-map comparison of regulatory elements in *HLA-C* alleles

Sequences homologous to HLA-C*05:01:01:01 and HLA-C*07:02:01:03 were identified in the NCBI nucleotide database using BLAST+ (v. 2.3.0) (ref. 58). Sequences containing the promoter and 5′UTR, exons 2–3, and 3′UTR regions were considered for the analysis. The region of the sequences aligned was as follows: promoter and 5′UTR region was −859 bp relative to the start codon of HLA-C*05 or −864 bp relative to the start codon of HLA-C*07; exons 2–3 region (including intron) length was 798 bases for the alignment, which included 792 bp for C*05 and 796 bp for C*07; the 3′UTR region included 541 bp after the stop codon in C*05 and C*07. For each complete sequence, we determined the *HLA-C* allele by aligning the individual sequence in the HLA-C IMGT data set^55^ to the query sequence and assigning the most similar allele. In some cases, sequences that had the same allele typing but a different accession number were obtained, and are shown in Supplementary Fig. 8 for completion. For display on the heat map, one representative sequence of each allele type was chosen. Pairwise alignments were performed with the Needleman–Wunsch algorithm in the EMBOSS package (v. 6.6.0.0) (ref. 59). Multiple sequence alignments were performed for each regulatory region (promoter and 5′UTR, exons 2–3, and 3′UTR) using MUSCLE (v3.8.31) (ref. 56) and phylogenetic trees were generated using MrBayes (v3.2.5) (ref. 57) with model parameters mentioned previously. To quantify the relative similarity between a *HLA-C* allele and HLA-C*05 and HLA-C*07 in a phylogenetic tree, we calculated a metric quantifying the phylogenetic distance between the query *HLA* allele and its relative distance to HLA-C*05 and HLA-C*07 in the specific tree. This was done in the following way: phylogenetic distances as branch lengths from the *HLA-C* allele to HLA-C*05 and to HLA-C*07 were extracted using the R package ape^60^, the similarity metric was then calculated as the ratio of the distance of the query *HLA-C* sequence to HLA-C*05 divided by the sum of the distances of the query *HLA-C* sequence to HLA-C*05 and HLA-C*07.

### Data availability

Structural information has been deposited in the Protein Data Bank^61^ under accession numbers 5VGD (HLA-C*05:01-SAE) and 5VGE (HLA-C*07:02-RYR). The mass spectrometry proteomics data have been deposited to the ProteomeXchange Consortium via the PRIDE^62^ partner repository with the data set identifier PXD006455. The data that support the findings of this study are available from the corresponding author on request.

## Additional information

**How to cite this article:** Kaur, G. *et al*. Structural and regulatory diversity shape HLA-C protein expression levels. *Nat. Commun.* **8,** 15924 doi: 10.1038/ncomms15924 (2017).

**Publisher’s note:** Springer Nature remains neutral with regard to jurisdictional claims in published maps and institutional affiliations.

## Supplementary Material

Supplementary Information

Supplementary Data 1
