# Supplementary material for: Structural and regulatory diversity shape HLA-C protein expression levels
Source: Nat Commun. 2017 Jun 26;8:15924. doi: 10.1038/ncomms15924 (PMC5490200; doi:10.1038/ncomms15924)
Supplement: Supplementary Information [file ncomms15924-s1.pdf]

Type of file: PDF
Title of file for HTML: Supplementary Information
Description: Supplementary Figures and Supplementary Tables.

Type of file: XLSX
Title of file for HTML: Supplementary Data 1
Description: Details of the specific peptides isolated from HLA-C*05:01 and HLA-C*07:02 molecules from transfected 721.221 cells.

Supplementary Figure 1

a

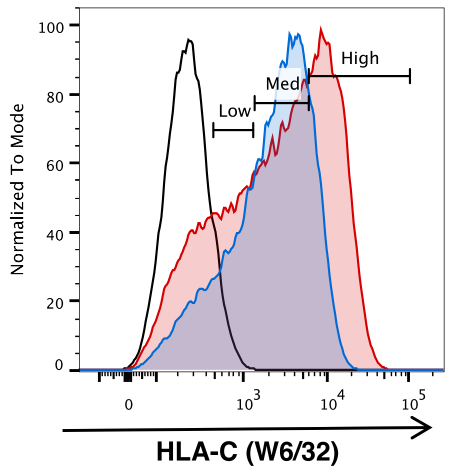

Genomic construct	% Low	% Med	% High
*C*05*	16.3	31.3	36.9
*C*07*	16.7	52.1	20.2

b

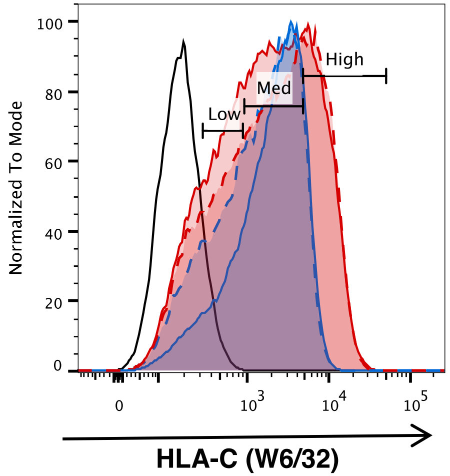

Promoter/5'UTR	Exon 1 – Intron 3	% Low	% Med	% High
*C*05*	*C*05*	19.6	41.6	29.1
*C*05*	*C*07*	19.6	55.8	14.5
*C*07*	*C*05*	17.7	39.5	33.3
*C*07*	*C*07*	12.8	61.8	20.1

Supplementary Fig. 1 - Differential expression of HLA-C*05 and HLA-C*07

(**a**) Representative cell surface expression of HLA-C on 721.221 cells transfected with the *C*05* and *C*07* genomic constructs. HLA-C (W6/32) staining is shown on GFP+ cells. Expression level is categorised as Low, Medium or High, and numbers denote percentage of positive cells in each gate. C*05 (red), C*07 (blue) and vector transfected cells (black) are shown. (**b**) Representative cell surface expression of HLA-C on 721.221 cells transfected with the *C*05* and *C*07* genomic constructs with or without the swapped promoter/5'UTR. HLA-C (W6/32) staining is shown on GFP+ cells. Expression level is categorised as Low, Medium or High, and numbers denote percentage of positive cells in each gate. Histogram colour coding is indicated in the Figure, black line represents vector-transfected cells.

Supplementary Figure 2

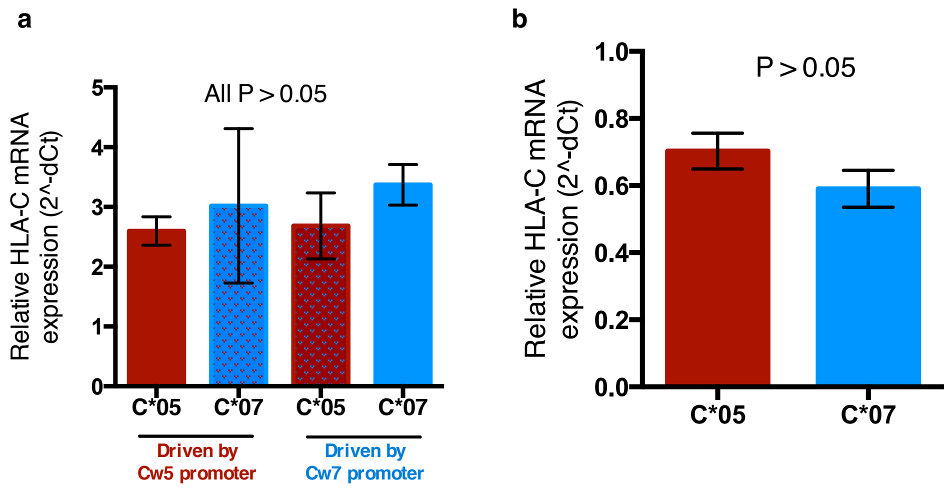

Supplementary Fig. 2 – Differential cell surface expression of HLA-C*05 and HLA-C*07 in transfected cells is not due to changes at the transcript level

(**a**) Relative HLA-C expression on *C*05* and *C*07* transfected 721.221 cells, determined using a QPCR assay in the common region of the constructs, and normalised to GFP expression in transfected cells. (**b**) Relative HLA-C expression in 721.221 cells transduced with the modified lentivirus expressing C*05 and C*07, using a QPCR assay in the common region of the constructs, and normalised to GFP expression in transduced cells. Mean ± S.E.M is depicted, (**a**) n=4-6, (**b**) n=7.

Supplementary Figure 3

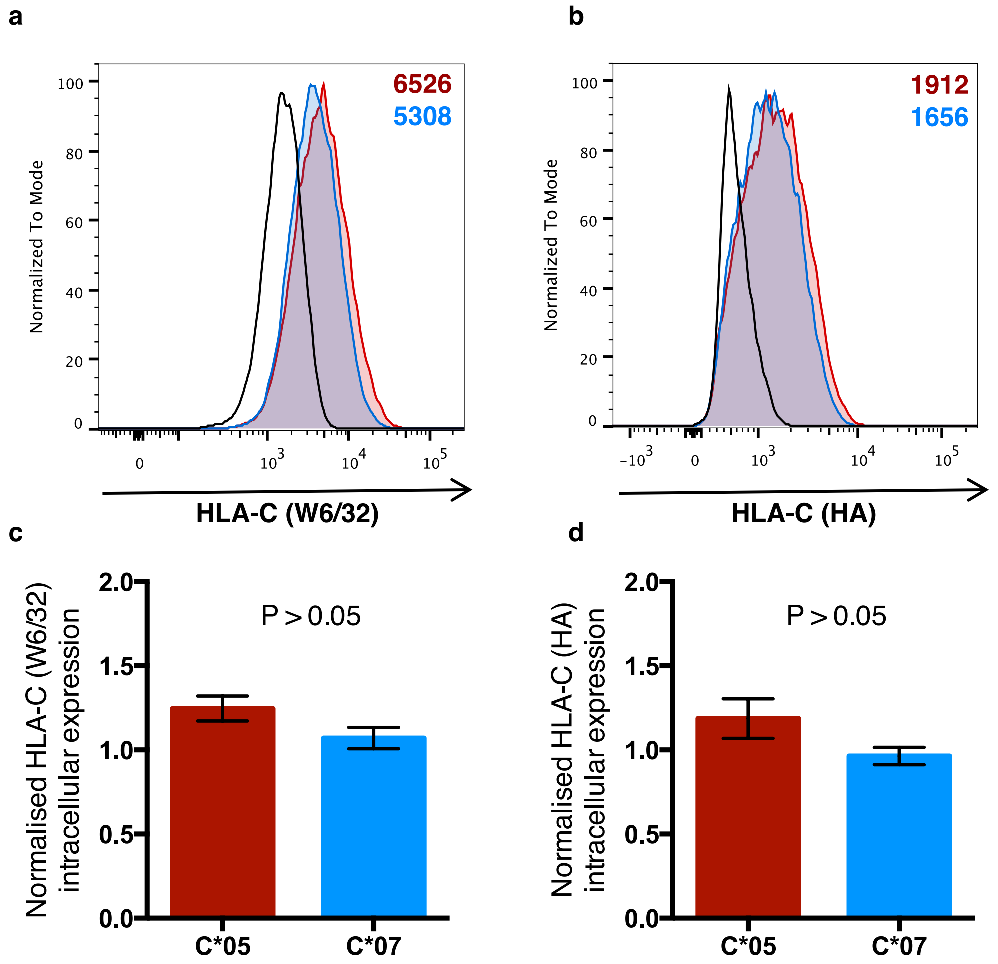

Supplementary Fig. 3 – Total HLA-C expression remains unchanged in lentivirus transduced C*05 and C*07 expressing cells

Representative total HLA-C staining performed after fixation and permeabilisation of 721.221 cells transduced with the lentiviral *C*05* and *C*07* constructs. (**a**) Total HLA-C (W6/32) staining and (**b**) HLA-C (HA) staining is shown on GFP+ cells. *C*05* (red), *C*07* (blue) and vector transduced cells (black) are shown, numbers denote MFI. (**c**) Normalised total HLA-C (W6/32) expression and (**d**) HLA-C (HA) expression on GFP+ *C*05* and *C*07* transduced 721.221 cells stained after fixation and permeabilisation of cells. MFI of W6/32 or HA/MFI of GFP, on the GFP+ population is plotted, and shown relative to *C*07* transduced cells. Mean ± S.E.M is depicted, (**a, c**) n=6, (**b, d**) n=5.

Supplementary Figure 4

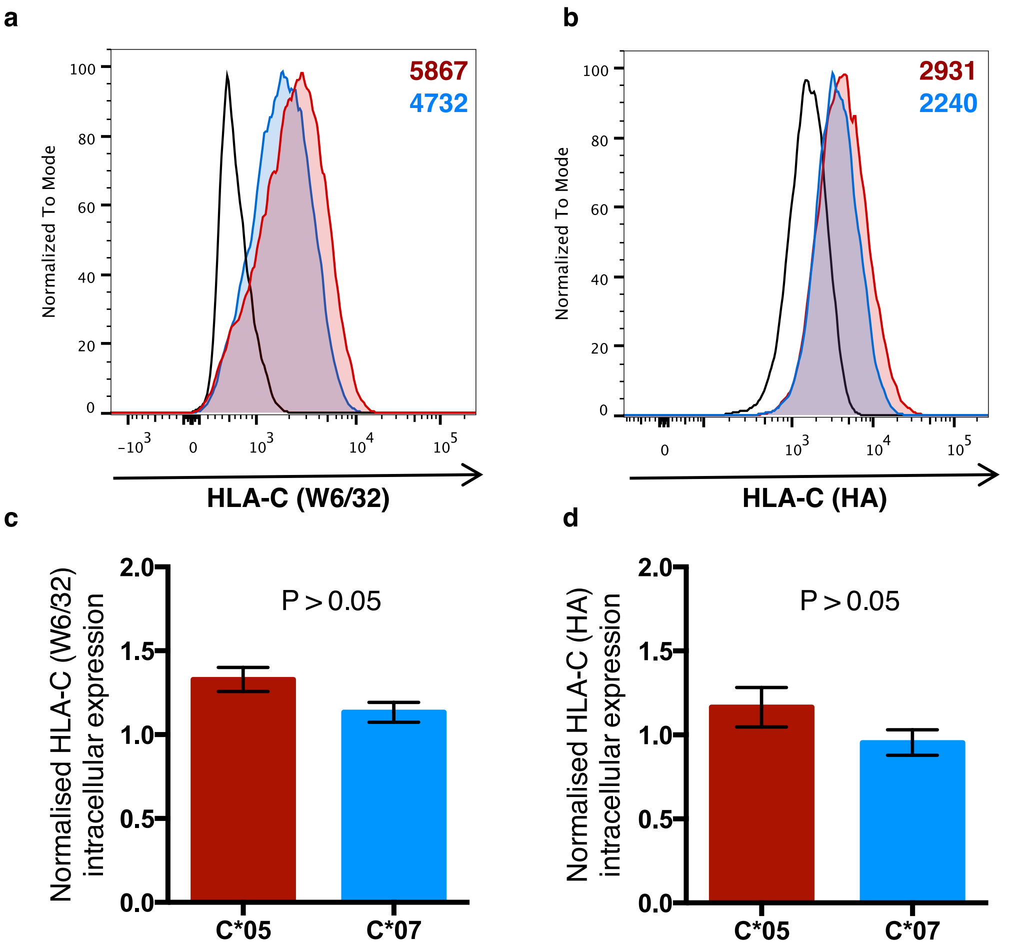

Supplementary Fig. 4 – Total HLA-C expression remains unchanged in modified lentivirus transduced C*05 and C*07 expressing cells

Representative total HLA-C staining performed after fixation and permeabilisation of 721.221 cells transduced with the modified lentiviral *C*05* and *C*07* constructs. (**a**) Total HLA-C (W6/32) staining and (**b**) HLA-C (HA) staining is shown on GFP+ cells. *C*05* (red), *C*07* (blue) and vector transduced cells (black) are shown, numbers denote MFI. (**c**) Normalised total HLA-C (W6/32) expression and (**d**) HLA-C (HA) expression on GFP+ *C*05* and *C*07* transduced 721.221 cells stained after fixation and permeabilisation of cells. MFI of W6/32 or HA/MFI of GFP, on the GFP+ population is plotted, and shown relative to *C*07* transduced cells. Mean ± S.E.M is depicted, (**a, c**) n=9, (**b, d**) n=6.

Supplementary Figure 5

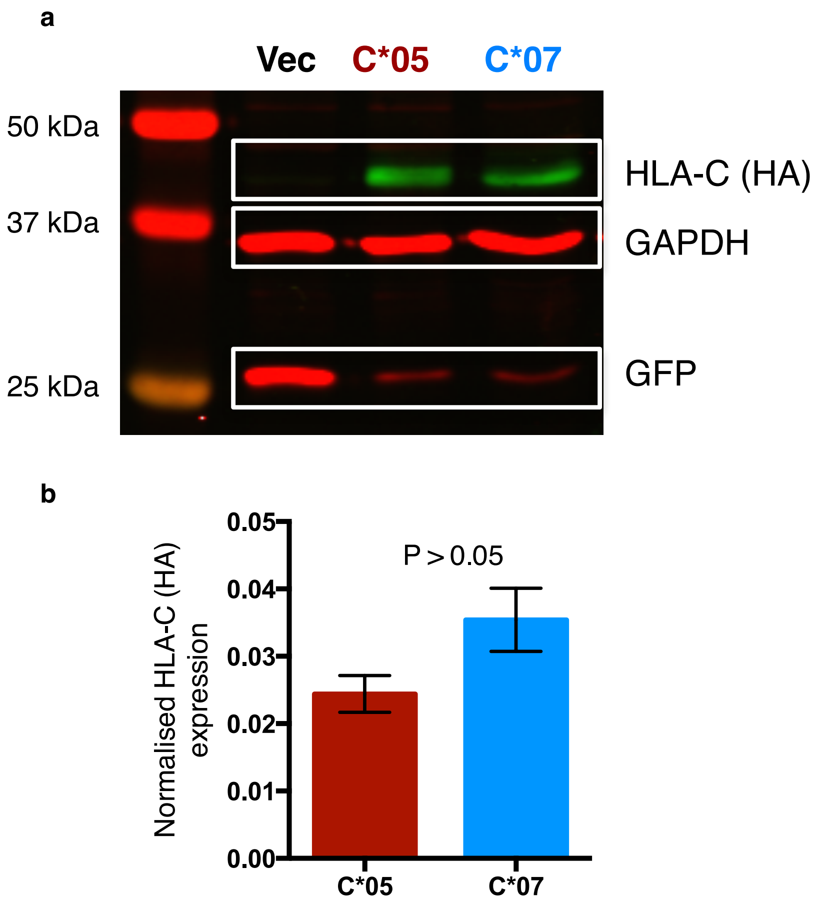

Supplementary Fig. 5 – Total protein levels of HLA-C in lentivirus transduced C*05 and C*07 expressing cells

(**a**) Total protein levels of HLA-C as assayed by immunoblotting using an anti-HA antibody on whole cell lysates from 721.221 cells transduced with lentiviral *C*05* and *C*07* constructs. (**b**) Quantification of total HLA-C protein levels relative to GAPDH and normalised for GFP expression in transduced cells. Mean ± S.E.M is depicted, n=6.

Supplementary Figure 6

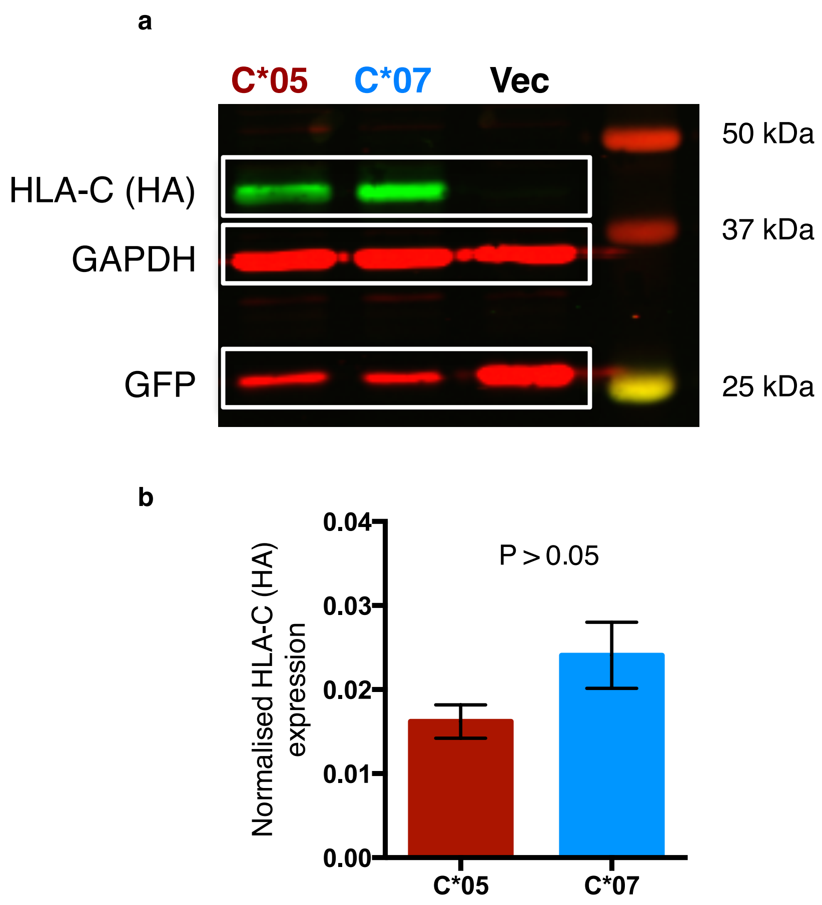

Supplementary Fig. 6 – Total protein levels of HLA-C in modified lentivirus transduced C*05 and C*07 expressing cells

(**a**) Total protein levels of HLA-C as assayed by immunoblotting using an anti-HA antibody on whole cell lysates from 721.221 cells transduced with modified lentiviral *C*05* and *C*07* constructs. (**b**) Quantification of total HLA-C protein levels relative to GAPDH and normalised for GFP expression in transduced cells. Mean ± S.E.M is depicted, n=7.

Supplementary Figure 7

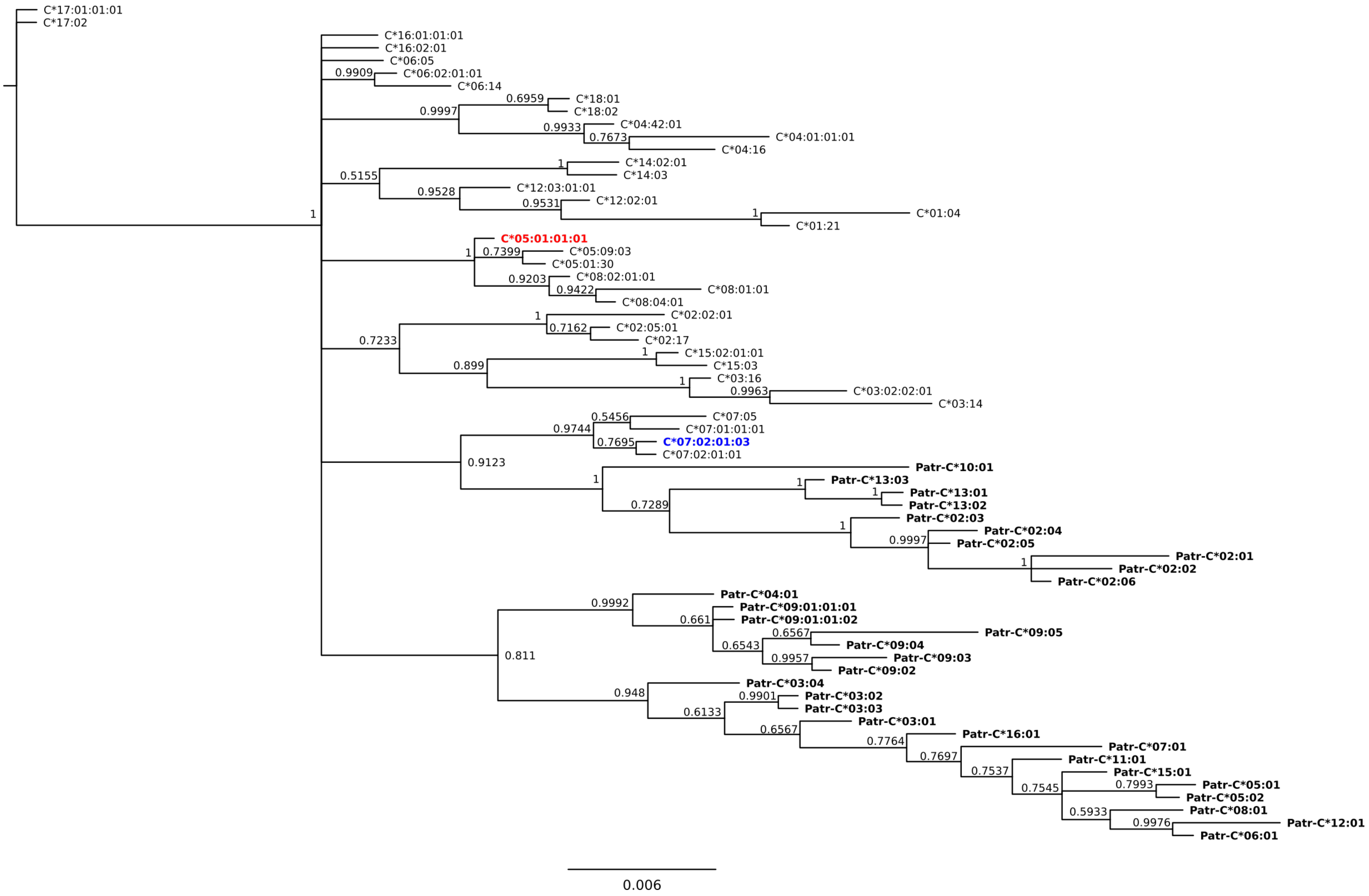

Supplementary Fig. 7 - Phylogenetic analysis of *HLA-C* and chimpanzee *MHC-C* sequences in the exons 2 and 3 region

HLA-C exon 2 and exon 3 sequences were aligned and used to infer phylogenetic relationship. Nodes are labelled with the estimated posterior for each split in the tree. The scale represents expected substitutions per site. *HLA-C*05:01:01:01* (red), *HLA-C*07:02:01:03* (blue), other *HLA-C* alleles (black) and chimpanzee *Patr-C* alleles (black bold) are shown.

Supplementary Figure 8

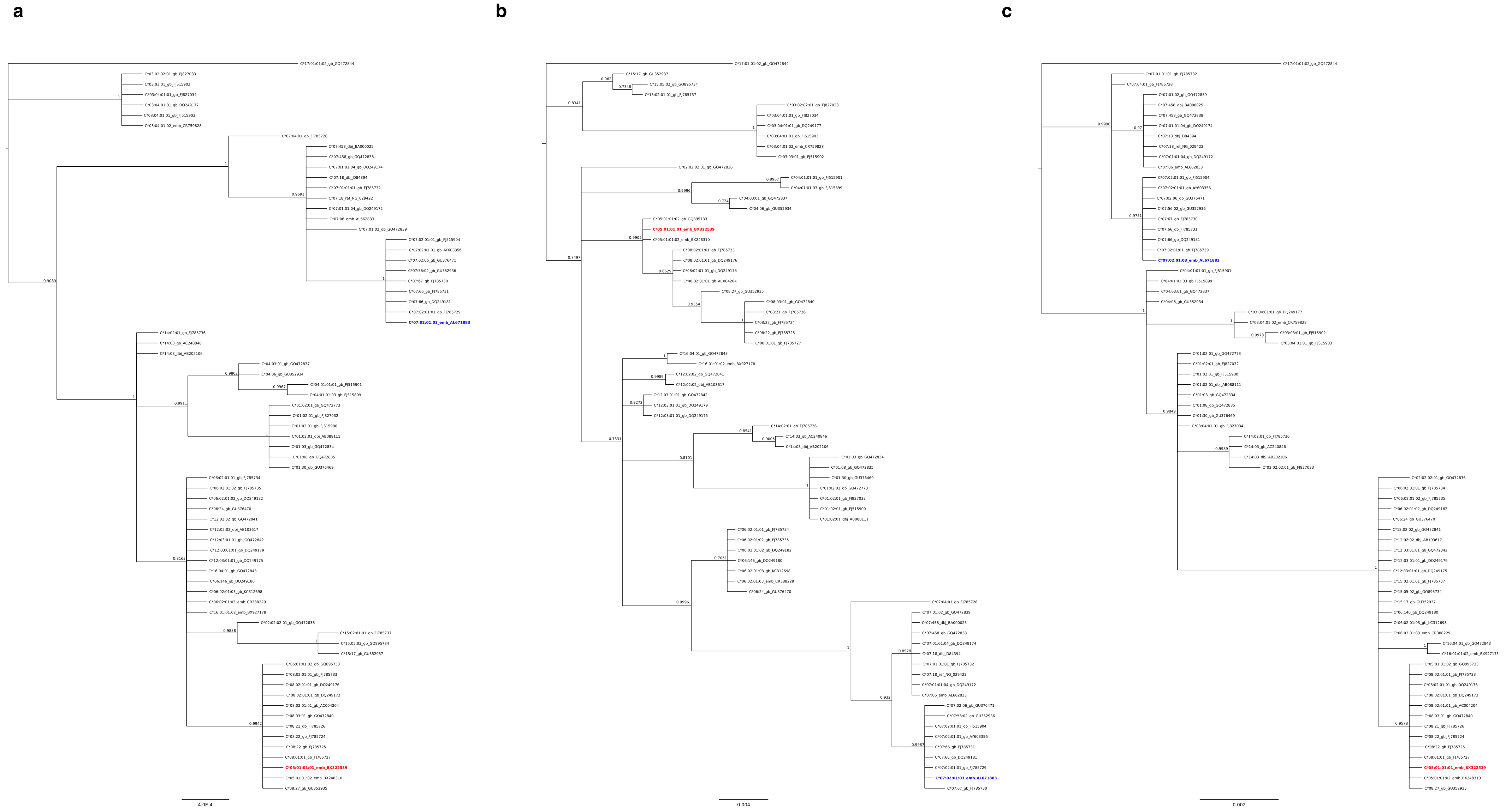

Supplementary Fig. 8 - Phylogenetic analysis of *HLA*-C alleles over three regulatory sites

Phylogenetic trees for (**a**) promoter and 5'UTR, (**b**) exon 2 to exon 3, and (**c**) 3'UTR region are shown. Construction of phylogenetic trees is detailed in the supplemental experimental methods. The scale represents expected substitutions per site. *HLA-C*05:01:01:01* (red), *HLA-C*07:02:01:03* (blue) and other *HLA-C* alleles (black) are shown.

SUPPLEMENTARY TABLE 1 - Data Collection and Refinement Statistics

**Data Collection Statistics**	HLA-C*05-SAE	HLA-C*07-RYR
Temperature	100K	100K
Space group	$P2_12_12_1$	$P6_5$
Cell Dimensions (a,b,c) (Å)	44.06, 80.54, 118.60	99.67, 99.67, 77.91
Resolution (Å)	47.74 – 2.30 (2.39 – 2.30)	43.16 – 2.60 (2.72 – 2.60)
Total number of observations	133256 (11942)	154533 (18546)
Number of unique observations	19225 (1744)	13665 (1657)
Multiplicity	6.9 (6.8)	11.3 (11.2)
Data completeness (%)	99.4 (94.5)	100.0 (99.9)
I/σ_I	11.5 (2.1)	14.8 (2.0)
$R_{pim}{}^{a}$ (%)	5.7 (38.7)	5.0 (46.3)
**Refinement Statistics**		
Non-hydrogen atoms		
Protein	3151	3135
Water	150	20
$R_{factor}{}^{b}$ (%)	22.01	16.32
$R_{free}{}^{b}$ (%)	29.72	22.08
Rms deviations from ideality		
Bond lengths (Å)	0.010	0.009
Bond angles (°)	1.300	1.384
Ramachandran plot (%)		
Allowed region	94	94
Generously allowed region	5	6
Disallowed region	1	0

[a] $R_{p.i.m} = \Sigma_{hkl} [1/(N-1)]^{1/2} \Sigma_i \mid I_{hkl,i} - \langle I_{hkl} \rangle \mid / \Sigma_{hkl} \langle I_{hkl} \rangle$

[b] $R_{factor} = \Sigma_{hkl} \mid\mid F_o \mid - \mid F_c \mid\mid / \Sigma_{hkl} \mid F_o \mid$ for all data except ≈ 5% which were used for R_{free} calculation

SUPPLEMENTARY TABLE 2 - Thermal stability of HLA-C*05 and HLA-C*07 complexes.

**Peptide**	**SAE**	**ITA**	**RYR**	**KYF**
**HLA-C*05 Tm (°C)**	51.5 ± 2.0	51.9 ± 2.4	52.2 ± 2.0	51.5 ± 1.5
**HLA-C*07 Tm (°C)**	45.0 ± 1.0	50.5 ± 0.5	54.5 ± 1.0	56.0 ± 1.0
